# Evidence inhibitory self‐control moderates effects of habit on complex but not simple health behaviors

**DOI:** 10.1111/aphw.12642

**Published:** 2024-12-22

**Authors:** Daniel J. Phipps, Martin S. Hagger, Kyra Hamilton

**Affiliations:** ^1^ Faculty of Sport and Health Sciences University of Jyvaskyla Jyvaskyla Finland; ^2^ School of Applied Psychology Griffith University Mt Gravatt Queensland Australia; ^3^ Health Sciences Research Institute Department of Psychological Sciences University of California Merced California USA

**Keywords:** behavioral automaticity, habit, health behavior, self‐control

## Abstract

Theoretically, self‐control can be considered as both a facilitator of habit development and a moderator of whether behavior occurs habitually. However, debate remains on the contexts in which such relationships are likely to occur. The current study tested whether self‐control, conceptualized into inhibitory and initiatory facets, would predict healthy behavior via habits or moderate the habit‐behavior relationship, and whether these effects differed across complex (bootcamp attendance *N* = 69, physical activity in pregnant women *N* = 115) and simple (flossing *N* = 254) behaviors. Three independent samples completed measures of self‐control and habit, followed by a prospective measure of behavior. Data were fitted to PLS‐SEM models. Inhibitory and initiatory self‐control predicted habit in all three samples, and habit in turn predicted each health behavior. Inhibitory self‐control only moderated the effect of habit in the bootcamp and physical activity samples. Initiatory self‐control did not moderate effects in any sample. Findings indicate that both initiatory and inhibitory self‐control are associated with habit. Further, as the moderating effect of inhibitory self‐control was only present in the complex behavior samples, results suggest the moderating effects of self‐control on the habit‐behavior relationship may be best represented by the effect of inhibiting competing cues from disrupting automatically activated behavioral sequences.

## INTRODUCTION

Given pervasive evidence of the link between behavior and health outcomes (Bloom et al., 2012), researchers and practitioners interested in the promotion of adaptive health outcomes through behavior change have placed increasing emphasis on the psychological determinants of health behavior. This is based on the assumption that psychological constructs represent the mental processes that mediate participation in health‐promoting behavior proposed in motivational and decision‐making theories, like temporal self‐regulation theory (Hall & Fong, 2007), reasoned action models (Fishbein & Ajzen, 2011), or dual‐process theories (Strack & Deutsch, 2004); and, that these psychological constructs are likely to be manipulable through techniques designed to do so (for examples of behavior change techniques see Abraham & Michie, 2008). It follows, therefore, that such determinants may signal potentially useful targets for behavioral interventions designed to promote the uptake and maintenance of health behaviors. Psychological research applying such theories has identified numerous determinants of health behavior. In particular, belief‐based constructs such as attitudes, social norms, self‐efficacy, and risk perception have been consistently associated with health behavior across contexts and populations (McEachan et al., 2011; Zhang et al., 2019). However, recognizing that continued participation in health behaviors may also arise from processes that do not involve elaborated cognition or deliberation (Bargh & Chartrand, 1999; Strack & Deutsch, 2004), researchers have become focused on determinants derived from dual‐process theories of action. That is, theories that represent non‐conscious processes that line up behavior beyond individuals' awareness and with little cognition or deliberation. Foremost among these has been the construct of habit (Gardner & Rebar, 2019; Hagger, 2020; Verplanken & Orbell, 2003).

Early approaches defined and conceptualized habit solely on behavioral terms and used proxy measures to capture it, such as frequency measures of prior behavior (Ouellette & Wood, 1998). Contemporary approaches conceptualize habit as a psychological construct comprising multiple defining characteristics beyond mere frequency such as experiencing automaticity, a lack or absence of awareness, effort, and deliberation when enacting the behavior, and dependency of acting on the presence of contingent conditions or ‘cues’ (Gardner, 2012; Verplanken & Aarts, 1999). Self‐report measures capturing these features have demonstrated consistent associations with health behavior (Gardner et al., 2011; Hagger et al., 2023; Phipps et al., 2020, 2021; van Bree et al., 2015), often with comparable effect sizes to those for intention or other social cognition, belief‐based constructs. These robust findings notwithstanding, meta‐analyses have identified considerable heterogeneity across studies in habit effects (Gardner et al., 2020, 2024; Hagger, 2018). Such heterogeneity has catalyzed interest in moderating conditions that could potentially determine the extent to which behaviors tend to be controlled by habit, represented as larger habit‐health behavior effects in modeling. Identifying such moderators may further elucidate the conditions conducive to habit formation and habitual behavior and have utility to those interested in developing intervention methods to promote health behaviors as habits.

One line of research offering considerable potential to identify moderators of habit effects on behavior has focused on intra‐individual conditions such as individual difference constructs or traits such as personality. In particular, researchers have identified the trait self‐control as a candidate moderator. Trait self‐control refers to an individual's general capacity to regulate their behavior, thoughts, and emotions indicated by a superior proficiency to alter or override impulsive or dominant responses (De Ridder et al., 2012). Individuals reporting ‘good’ trait self‐control exhibit good self‐regulatory capacity affording an advantage toward engaging in goal‐directed behavioral pursuit and negating potentially distracting or derailing contingencies (Gottfredson & Hirschi, 1990). Unsurprisingly, trait self‐control has been consistently associated with health behavior participation (De Ridder et al., 2012; Wood, 2016). Although effect sizes for trait self‐control tend to be small (Hagger & Hamilton, 2024), this is consistent with other research examining the effects of domain‐general intra‐personal constructs on health behavior (Bogg & Roberts, 2004).

Although the majority of trait self‐control research focuses on self‐control as an overall construct, some debates in the field have argued self‐control to be a multi‐faceted construct (de Ridder et al., 2011; Duckworth et al., 2016; Hagger et al., 2021). While there is some variability in the exact facets of self‐control, such multi‐factor conceptualizations of self‐control generally suggest that the construct comprises a component that focuses on the capacity to actively engage in goal‐directed behavior; thus, a motivational or approach tendency (‘initiatory self‐control’), and a component that focuses on the capacity to inhibit impulses and resist temptations and distractions (‘inhibitory self‐control’). Together, these two components of self‐control likely afford individuals an advantage when pursuing goal‐directed behaviors (de Ridder et al., 2011). For example, initiatory self‐control may encourage healthy behavior via its effects on the formation of pro‐health beliefs (Hagger et al., 2018) or as a function of those with high initiatory self‐control structuring their environment as to make easier their target behavior (Duckworth et al., 2014; Stojanovic & Wood, 2024), while those with higher inhibitory self‐control may be more likely to enact their target behavior when the opportunity arises through the ability to overcome competing impulses or temptations, allowing for an increased frequency of the behavior in the presence of activating cues. Thus, the application of both inhibitory and initiatory self‐control should lead individuals to engage in consistent practice of the behavior in stable contexts – precisely the conditions conducive to habit formation, which in turn may be theoretically expected to manifest as sustained participation in the behavior. Consistent with this notion, research indicates that habit mediates the relationship between self‐control and health behaviors (Adriaanse et al., 2014; McCarthy et al., 2017; Pfeffer & Strobach, 2018).

It may also be the case that trait self‐control serves as a moderator of the effect of habit on behavior (Charlesworth et al., 2022; Honkanen et al., 2012; Phipps et al., 2023). For example, a recent study indicated that trait self‐control moderated habit‐health behavior effects *negatively* (Conner et al., 2023), as the effect of habit on unhealthy behavior was smaller in those with high self‐control. The authors indicated that the pattern of findings may have been because self‐control affords the same advantages outlined earlier; better capacity to override impulses and break unwanted habits. The negative moderation explanation makes conceptual sense in the context of health risk behaviors (e.g., excess alcohol consumption, sedentary behavior) given that better self‐control should afford individuals with better capacity to override impulses and not succumb to ‘bad’ habits. However, in the context of health‐promoting behaviors (e.g., physical activity, bootcamp attendance, dental flossing), we speculate that habit will moderate the habit‐health behavior relationship *positively*, such that the relationship between healthy habits and behavior will be larger in those reporting higher levels of trait self‐control. For example, those high in inhibitory self‐control may be more likely to complete a habitually initiated healthy behavior, even in the face of temptations or competing impulses. Alternatively, the use of more *cold* or initiatory self‐control skills has also been associated with environmental restructuring (Duckworth et al., 2014, 2016). That is, those high in initiatory self‐control may be more likely to make use of their habits in the pursuit of desirable behavior, for example by altering their context to increase the likelihood of encountering a healthy behavior triggering cue or reducing the effort or motivation required for behavioral enaction (Stojanovic & Wood, 2024).

Beyond the potential effects of habit as a mediator of the self‐control–behavior relationship and of self‐control as a candidate moderator of the habit–behavior relationship, it is also possible these patterns of effects are dependent on situational factors specific to a given behavior, such as its complexity. Theory and research on habit indicate that complex behaviors comprising multiple sub‐actions that demand considerable strategizing and organization to perform are often more difficult to be developed as habits (Gardner et al., 2020; Hagger, 2018; Lally et al., 2010). In contrast, those behaviors that comprise single actions or very few sub‐actions demand less executive co‐ordination and are therefore more conducive to habit formation. This does not mean that complex behaviors cannot be, to some degree, developed as habits – with sufficient repetition in stable contexts many of the sub‐actions may with time ultimately come to be developed as habits or, at least, require less conscious control. For example, if sufficient numbers of the sub‐actions ultimately fall under automatic control, the net effect may be that the behavior is formed as a habit (Hagger, 2020).

Further, some researchers have suggested that it is the initiation or decision to enact the sequence of sub‐actions comprising complex behaviors that may become habitual rather than the behavior itself (Gardner et al., 2020; Phillips & Gardner, 2016; Rhodes & Rebar, 2018). For example, one may automatically decide to go for a jog and begin looking for shoes and sportswear, but the process of getting ready to go on a jog, planning a route, and beginning the jog involves considerable strategizing and co‐ordination and is unlikely to be enacted without extensive deliberation. Accordingly, researchers have observed larger habit effects on behavior, including for health behaviors, when the behavior is considered low in complexity relative to behaviors categorized as higher in complexity (Hagger et al., 2023).

Individuals with ‘good’ trait self‐control, however, may be afforded an advantage when it comes to developing and enacting more complex behaviors as habits. For example, the superior self‐regulatory capacities that ‘good’ initiatory self‐control confers may increase individuals' propensity to form habits even for complex behaviors because they are more able to engage in the behavior despite the fact it may be more effortful or time‐consuming. Similarly, as habit formation for complex behaviors likely requires an intrinsic reward, the formation of habits for complex behaviors is contingent on the completion of sub‐actions resulting in the desired behavior triggering the intrinsic reward (Phillips & Mullan, 2023). Thus, the inhibitory self‐control skills that suppress competing impulses or temptations may aid in habit formation as those high in inhibitory self‐control are able to consistently perform the sufficient sub‐actions of the behavior more effectively. Further, even when complex behaviors become increasingly habitual, they still typically include more sub‐actions than simpler behaviors, some of which may require substitution or conscious input (Hagger, 2020; Phillips & Mullan, 2023). It is plausible that this increased complexity and number of sub‐actions may leave such behaviors more at risk of distraction via competing impulses or more immediately tempting alternative behaviors. While speculative, this is reflected in recent evidence showing the use of self‐regulation skills in behavioral enactment for complex behaviors was high regardless of whether that behavior was enacted habitually or as the consequence of considered decision‐making (Saunders & More, 2024). Thus, it is possible that the impact of self‐control on habit and habitual enaction may vary in complexity as compared to simple behaviors, although this effect has been seldom tested to date.

## THE CURRENT STUDY

In the current study, we aimed to test hypotheses relating to the effects of a two‐factor conceptualization of trait self‐control on the habit‐behavior relationship in health behaviors of varying complexity. First, we tested whether the effects of initiatory and inhibitory self‐control on health behavior would be mediated by the habit construct in each behavior. Specifically, we expected each form of self‐control would be associated with the habit (H1) and habit, in turn, would be associated with more frequent behavior (H2), although some residual direct effect of self‐control was expected to remain (H3). Thus, we predicted indirect effects of both components of trait self‐control on each health behavior via habit (H4), although we expected larger effects for the inhibitory component consistent with prior research. Alongside this, we also tested the extent to which these self‐control components moderated the effects of the habit construct on health behavior, such that higher self‐control would be associated with a larger habit‐behavior relationship (H5).

Beyond the tested model, we also make several hypotheses regarding differences between behaviors categorized as complex (i.e., behavior for which the execution requires multiple sub‐actions or additional cognitive processing) and simple (i.e., behaviors requiring fewer sub‐actions or less cognitive processing). While we predicted that both inhibitory and initiatory self‐control would serve to predict habit and to moderate the habit‐behavior effect, we expected larger effects when the behavior was considered higher in complexity as opposed to when the behavior was classified as lower in complexity (H6). We tested our hypotheses in three independent samples in two behaviors classified as high in complexity ‐ physical activity and attending bootcamp classes; and one behavior considered less complex ‐ dental flossing, based on prior research (Hagger et al., 2023).

## METHODS

### Design and procedure

Study hypotheses were tested in three samples (bootcamp attendance, physical activity, and flossing behaviors), adopting two‐wave correlational survey designs. At baseline, eligible participants in each sample were presented with details on the purpose of the research and informed consent forms. Specifically, participants were informed that the research aimed to measure people's current behaviors and habits as well as their perceived beliefs in relation to the target behavior, but did not require them to actively change or alter their behavior. No particular level of habit strength was required in order to be eligible for the study. After providing informed consent, participants then completed measures of the psychological constructs, habit, and trait‐self‐control on the initial data collection occasion (T1). Data on each target behavior was administered on a second, follow‐up occasion (T2). All materials were hosted using the online Qualtrics platform.

### Participants

#### Sample 1 – Bootcamp attendance

In Sample 1 attendees to commercially organized ‘bootcamp’ exercise sessions were recruited via questionnaires distributed by class organizers. A total of 158 completed T1 measures. Four weeks later, 69 participants provided follow‐up behavioral data on their bootcamp attendance (*M* age = 35.84, *SD* = 11.47, 16 male, 53 female). Participants who did not complete the behavioral follow‐up did not differ from the final sample on time one score for inhibitory self‐control, initiatory self‐control, and habit (Wilk's Lambda = .97, *F*[3, 154] = 1.78, *p* = .153), age (*t*[156] = 1.37, *p =* .173, *d* = .20), or gender (χ^2^[1] = 2.38, *p* = .123).

#### Sample 2 – physical activity

At T1 the second sample, pregnant women, were recruited via face‐to‐face contact at mother/baby groups and general practice surgeries, along with advertisements at antenatal classes, childcare centers, and on social media. A total of 207 completed surveys on the initial data collection occasion, with 115 providing valid follow‐up behavioral data one week later (*M* age = 30.59, *SD* = 4.40). Participants who completed the follow‐up survey did not differ from those who did not on scores on inhibitory self‐control, initiatory self‐control, and habit (Wilk's Lambda = .97, *F*[3, 203] = 2.45, *p* = .065) or age (*t*[205] = 1.64, *p =* .102, *d* = .23).

#### Sample 3 – dental flossing

In the third sample, undergraduate students were recruited from the participant pool at a major Australian university in exchange for course credit. A total of 629 completed T1 measures. One week later, 254 participants returned to provide a measure of flossing behavior (*M* age = 22.23, *SD* = 6.40, 52 male, 202 female). Participants who did not complete T2 behavior measures did not differ from the final sample on inhibitory self‐control, initiatory self‐control, and habit at time 1 (Wilk's Lambda = .98, *F*[3, 621] = 0.48, *p* = .697) or gender (χ^2^[1] = 0.32, *p* = .569). However, participants who completed T2 were older than those who did not (*t*[626] = 4.33, *p* < .001, *d* = .35).

### Measures

#### The self‐report behavioral automaticity index (T1)

Habit was measured as behavioral automaticity in all samples using the self‐report behavioral automaticity index (Gardner et al., 2012).^1^ Items were scored on 7‐point Likert scales (1 = strongly disagree and 7 = strongly agree). Example items for each behavior include “Performing the recommended level of physical activity is something I do automatically”, “Attending bootcamp is something I do automatically”, and “Flossing is something I do automatically”.

#### Brief self‐control scale (T1)

Self‐control was assessed using the brief self‐control scale (Tangney et al., 2004) segregated into inhibitory and initiatory components consistent with a two‐factor model of trait self‐control (de Ridder et al., 2011). Six inhibitory self‐control items assessed participants' capacity to complete a goal in the presence of competing alternatives (e.g. “*I am good at resisting temptation*”), and four initiatory self‐control items referenced a participants' capacity to actively engage in goal‐directed behavior to achieve outcomes (e.g. “*I am able to work effectively towards long‐term goals*”). Items were scored on 5‐point Likert scales (1 = strongly disagree and 5 = strongly agree).

#### Bootcamp attendance (T2)

Bootcamp attendance in sample 1 was measured using two self‐report items targeting the frequency of bootcamp attendance in the previous month (e.g. “*Think about the past four weeks. In general, how often did you attend bootcamp?”)*. Items were scored on 7‐point Likert scales (1 = never and 7 = always).

#### Physical activity (T2)

In sample 2, self‐reported participation in physical activity was assessed using Godin and Shephard's leisure‐time physical activity questionnaire (Godin, 2011). Specifically, participants were asked to provide how often they engaged in strenuous, moderate, and light physical activity each week. Responses to strenuous, moderate, and light physical activity were multiplied by 9, 5, and 3 respectively, to create a total score for the measure.

#### Dental flossing behavior (T2)

Dental flossing behavior in sample 3 was assessed using two self‐report items targeting the frequency of dental flossing in the past week (e.g. “*In the last week, how often did you floss?*”). Items were scored on 7‐point scales (1 = never and 7 = very often).

### Data analysis

Data were analyzed using partial least squared structural equation modeling in WarpPLS 6.0 (Kock, 2018). Survey items for each scale were used to indicate latent variables representing each construct or behavioral dependent variable. Standard errors were calculated using the stable method (Kock, 2018). For each model, the fit was assessed using the Tenenhaus goodness‐of‐fit index (GoF, Acceptable if > .25 assuming a medium effect size), average variance inflation factor (AVIF, acceptable if < 5), Sympson's paradox ratio (SPR, acceptable if > .7), and *R* ^2^ contribution ratio (acceptable if > .9) (Kock, 2018; Tenenhaus et al., 2004). Significant moderation effects were probed using bootstrapped median‐split simple slopes analysis. Following PLS‐SEM recommendations (Hair et al., 2017; Jhantasana, 2023), a sample of 70 or more was required to achieve a power of .80 for the overall model, assuming a medium‐sized effect (*R* ^2^ = .25) and an alpha level of .05. Further, assessing the power of individual regression pathways, a minimum sample of 55 was required to detect regression effects with a medium‐sized effect (*f* ^*2*^ = .15) at a power level of .80 (Faul et al., 2007). In addition to model tests, we also compared the strength of model parameters between samples using unequal variance assumed *t*‐tests as per Schenker and Gentleman (2001).

## RESULTS

**FIGURE 1:**
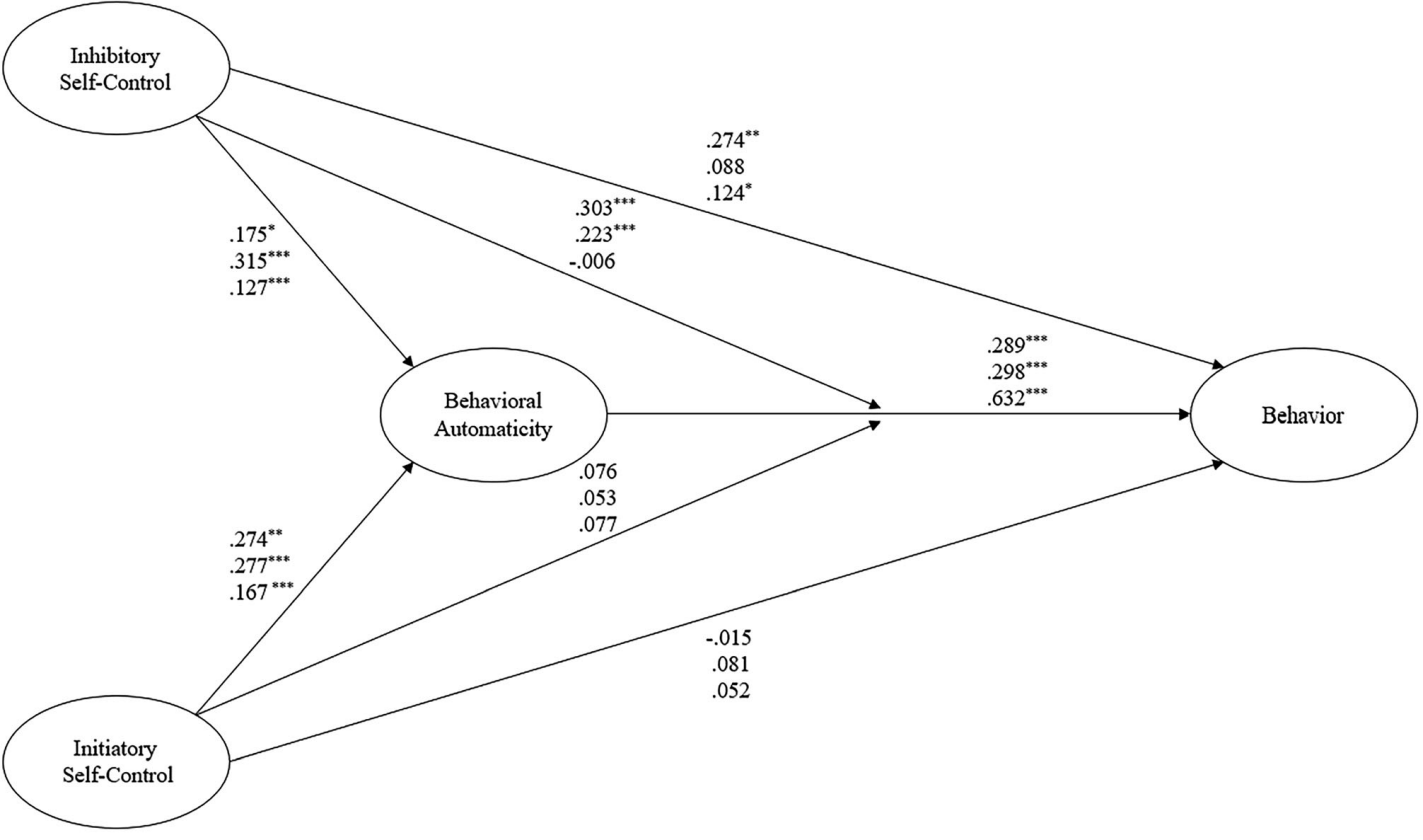
The hypothesised model of self‐control, habit, and health behaviour in physical activity, Bootcamp attendance, and flossing. *Note*: * *p* < .05, ** *p* < .01, *** *p* < .001. Coefficients on the top line refer to the physical activity sample. Coefficients on the middle line refer to the bootcamp sample. Coefficients on the lower line refer to the flossing sample.

Latent variable intercorrelations means and standard deviations, and reliability statistics for the Bootcamp attendance, physical activity, and dental flossing samples are presented in Table 1. Standardized parameter estimates for the hypothesized model effects in each sample are presented in Table 2 and Figure 1. The model exhibited a good fit with the data in all three samples and accounted for nontrivial variance in each behavior (Bootcamp Attendance GoF = .355, *R* ^*2*^ = .37, AVIF = 1.38, SPR = 0.86, RSCR = 0.99; Physical Activity GoF = .339, *R* ^2^ = .23, AVIF = 1.19, SPR = 1.00, RSCR = 1.00; Flossing GoF = .374, *R* ^2^ = .46, AVIF = 1.16, SPR = 1.00, RSCR = 1.00). We found the expected (H1) non‐zero effects of initiatory and inhibitory self‐control on habit, and of habit on behavior (H2) in all samples with small‐to‐medium effect sizes. Direct effects of self‐control on behavior were partially in line with expectations (H3), as inhibitory self‐control had a small, non‐zero direct effect on behavior in the bootcamp and flossing samples, but confidence intervals for this effect in the physical activity sample just encompassed zero as a potential value. By contrast, the direct effect of initiatory self‐control on behavior was not significantly different from zero in any of the samples.

**TABLE 1:** Correlations between variables in the model predicting boot camp attendance, physical activity, and flossing behavior.

	1	2	3	4	*M*	*SD*	α
Bootcamp attendance							
1. Inhibitory self‐control	‐				2.96	0.65	.61
2. Initiatory self‐control	.574^***^	‐			3.57	0.69	.48
3. Behavioral automaticity	.273*	.339^**^	‐		5.49	1.69	.94
4. Behavior	.334*	.178	.433^***^	‐	4.83	1.95	.94
Physical activity							
1. Inhibitory self‐control	‐				3.40	0.62	.69
2. Initiatory self‐control	.620^***^	‐			3.86	0.58	.48
3. Behavioral automaticity	.399^***^	.370^***^	‐		4.22	1.73	.94
4. Behavior	.165	.246^**^	.364^***^	‐	39.30	20.63	‐
Flossing							
1. Inhibitory self‐control	‐				2.94	0.69	.71
2. Initiatory self‐control	.510^***^	‐			3.41	0.69	.65
3. Behavioral automaticity	.157*	.156*	‐		2.41	1.77	.98
4. Behavior	.026	.025	.641^***^	‐	3.78	2.05	.96

*Note*: * *p* < .05, ** *p* < .01, *** *p* < .001.

**TABLE 2:** Path estimates for the models predicting bootcamp attendance, physical activity, and flossing behavior.

	Bootcamp attendance		Physical activity		Flossing behavior	
Path	β	*p*	β	*p*	β	*p*
Direct effects						
Inhibitory self‐control → behavioral automaticity	.175	.024	.315	<.001	.127	.009
Inhibitory self‐control → behavior	.274	.001	.088	.113	.124	.010
Initiatory self‐control → behavioral automaticity	.274	.001	.277	<.001	.167	<.001
Initiatory self‐control → behavior	−.015	.431	.081	.133	.086	.052
Behavioral automaticity → behavior	.289	<.001	.298	<.001	.632	<.001
Moderation effects						
Inhibitory self‐control by behavioral automaticity → behavior	.303	<.001	.223	<.001	−.006	.451
Initiatory self‐control by behavioral automaticity → behavior	.076	.192	.053	.235	.077	.073
Indirect effects						
Inhibitory self‐control → behavioral automaticity → behavior	.051	.206	.094	.034	.080	.017
Initiatory self‐control → behavioral automaticity → behavior	.079	.100	.083	.054	.106	.003
Total effects						
Inhibitory self‐control → behavior	.325	<.001	.175	.009	.204	<.001
Initiatory self‐control → behavior	.064	.231	.171	.010	.192	<.001

With respect to our hypothesized indirect effects (H4), habit mediated the effect of inhibitory self‐control on behavior in the physical activity and flossing samples, but not in the bootcamp attendance sample. Similarly, initiatory self‐control predicted dental flossing behavior indirectly through habit, while the effect of initiatory self‐control on behavior was in the expected positive direction but was no different from zero. As predicted (H5), inhibitory self‐control moderated the effect of habit on behavior positively in both the bootcamp attendance and physical activity samples, behaviors classed as high in complexity, such that the effect of habit was larger for those high in inhibitory self‐control (bootcamp β = .540, *SD* = .204, physical activity β = .274, *SD* = .111) than those with low inhibitory self‐control (bootcamp β = .299, *SD =* .185, physical activity β = .226, *SD* = .091). However, against expectations, inhibitory self‐control was not found to moderate the habit‐behavior relationship in the dental flossing sample, and initiatory self‐control did not moderate the habit‐behavior relationship in any sample.

In the comparison of effect sizes between samples (H6), we observed no statistically significant differences in parameter estimates between the physical activity and bootcamp samples (all *p*s > .102). Regarding direct effects, inhibitory self‐control had a larger effect on habit in the physical activity sample compared to the flossing sample (*p* = .037, *d* = .27), and the effect of behavioral automaticity on behavior was larger in the flossing sample as compared to both the physical activity (*p* < .001, *d* = .48) and bootcamp sample (*p* = .001, *d* = .61). Further, the moderating effect of inhibitory self‐control on the habit behavior relationship was more pronounced in the physical activity (*p* = .011, *d* = .33) and bootcamp samples (*p* = .003, *d* = .55), compared to the flossing sample. No other statistically significant effects were observed (all comparisons are available in online supplementary materials, Appendix C).

## DISCUSSION

The current study aimed to test a number of theory‐stipulated effects of trait self‐control on habit and health behavior. Specifically, we tested the direct effects of each construct on behavior, and, importantly whether trait self‐control effects on behavior were mediated by habit and trait‐self‐control moderated the habit‐behavior relationship in three health‐related behaviors. In addition, we aimed to evaluate whether the proposed pattern of effects varied according to the level of complexity of the target behavior. Finally, we also made the distinction between initiatory and inhibitory components of trait‐self‐control in our analysis consistent with a two‐factor model of trait self‐control. We observed direct effects of both trait self‐control components on habit, and of habit on behavior, in all samples. Further, we found indirect effects of trait self‐control on behavior mediated by habit in the physical activity and bootcamp samples. Consistent with our moderation hypotheses, inhibitory self‐control moderated the habit‐behavior relationship in the physical activity and bootcamp attendance samples, but not in the dental flossing sample. Initiatory self‐control did not moderate the habit‐behavior pathway in any sample.

The consistent effects of both inhibitory and initiatory self‐control on habit across samples corroborate predictions of the theory on habit and align with our first hypotheses. While the study sample potentially lacks the power to make definitive assertions as to the nature of this effect, one plausible theoretical explanation for those higher in self‐control reporting stronger habits may be that the effective capacities for self‐regulation afforded to those with higher trait self‐control are implicated in the development of healthy habits (Galla & Duckworth, 2015; Stojanovic & Wood, 2024). For example, individuals with initiatory self‐control are likely to possess a superior capacity to pro‐actively engage in the kind of consistent, repetitive goal‐directed behavior conducive to habit development, most likely because they have better capacity to apply self‐regulatory skills like identifying salient goals and subgoals and strategizing to form behavioral routines, and a functionally restructuring their environment so as to manage derailing or distracting contingencies (Carden & Wood, 2018). Inhibitory self‐control on the other hand likely affects habit development by minimizing the role of competing impulses, resolving goal conflicts, and promoting effective emotion regulation (Carden & Wood, 2018; Hagger et al., 2018), all of which are likely to be of importance in the early stages of habit formation (Carden & Wood, 2018). Thus, although we cannot make definitive assertions due to the correlational design employed, current findings are consistent with what may be expected from the notion that habit formation and maintenance represents a key mechanism by which trait self‐control may elicit healthy behavior (De Ridder et al., 2012; Stojanovic & Wood, 2024). However, this requires testing in longitudinal designs to confirm.

We also hypothesized that both initiatory and inhibitory self‐control components would moderate the effects of habit on health behavior, in line with prior theory and research (Adriaanse et al., 2014; Conner et al., 2023; Pfeffer & Strobach, 2018). Our hypothesis was supported in two of the three behaviors insofar as the inhibitory component moderated the habit‐behavior relationship for bootcamp and physical activity samples positively, but no moderating effect for dental flossing was observed. By contrast, we found no moderating effects for the initiatory component. While speculative, this aligns with recent evidence that individuals still report the use of self‐regulatory strategies even when a behavior is habitually initiated (Saunders & More, 2024), and that the effect of habit on behavior is weaker in individuals who hold implicit attitudes that run in contrast to their habit (Conner & Norman, 2005; Phipps et al., 2021), potentially representing the effect of distracting impulses. In combination, this evidence alongside the current results may indicate that regardless of whether a pro‐health behavior is performed as the result of conscious decision‐making or an automatically activated habit, self‐control may still increase the likelihood of behavior by protecting the desired sequence of sub‐actions from being derailed by competing impulses, temptations, or distractions. In contrast, the presented findings indicate that while initiatory self‐control is associated with habit strength, potentially due to its role in aiding in the formation of habit, these initiatory self‐control skills are not impactful in the likelihood of habits being reflected in behavioral frequency once a habit is formed.

In terms of differences in direct effects between behaviors, we observed few non‐zero differences between samples in the effects of each component of self‐control on habit, as while inhibitory self‐control had a larger relationship with habit in the physical activity sample, no other significant differences were observed. Such a finding runs in contrast to our expectation that self‐control may be a more important factor in habit formation for complex behaviors, as forming habits for more complex behaviors, such as bootcamp attendance and physical activity behaviors, is likely to require greater investment in terms of effort and time than less complex behaviors like dental flossing, and thus, was speculated to be more contingent on self‐control. However, current data indicates this may not be the case, or this relationship may have been too small to detect consistently in the current data.

Regarding the prediction of behavior between samples, the habit behavior relationship was notably larger in the flossing sample as compared to the physical activity and bootcamp samples. Such a finding is in line with meta‐analysis which suggests behaviors that require fewer steps or less cognitive elaboration to enact are more likely to fall under the control of habit as compared to complex behaviors (Hagger et al., 2023). However, for the moderating effect of inhibitory self‐control on habit, the lack of an effect for the dental flossing behavior stands in contrast to those found for the bootcamp attendance and physical activity behaviors. A potential explanation for this discrepancy may lie in the relative complexity of the behaviors – for simple behaviors individuals do not need to rely on these inhibitory capacities to enact the habitual behavior, whereas in complex behaviors, the ability to resist temptations or distractions is important to prevent the sequence of sub‐actions being derailed. This is again speculative but does align with research that the deployment of self‐regulation occurs equally in complex behaviors regardless of whether the behavior is habitual or not (Saunders & More, 2024), while simple habitual behaviors do not require the use of self‐regulatory strategies. We look to future research to verify this possibility, for example, studies in which behavioral complexity and self‐control skills are systematically manipulated. This may be achieved by, for example, employing novel behaviors that vary in complexity and training individuals in self‐control capacities, particularly inhibitory control capacities (e.g., Allom et al., 2016; Friese et al., 2016).

### Implications, limitations, and future directions

The current study has notable strengths including testing novel hypotheses relating to the role of key components of self‐control on habit formation across multiple behaviors varying in complexity. Findings have implications for elucidating the mechanisms by which self‐control and habit relate to health behavior. However, we should also identify some key limitations in light of which these findings should be interpreted. First, the correlational design of the current study precludes inferences of direction or cause in the reported effects– such inferences are derived solely from theory, not the data. For example, we hypothesized based on habit theory that self‐control would be associated with habits due to its role in promoting repeated healthy behaviors. Yet, while this association was reflected in our data, we are unable to definitively assert that the observed association is causal or directional due to the prospective correlational design employed. Future research may seek to employ more intensive certain types of longitudinal study designs, such as ecological momentary assessment methodology that may indicate processes of habit development through sampling sequences of experiences or certain implementations of cross‐lagged panel designs, that may assist in inferring direction through cross‐lagged effects among self‐control, habit, and behavior while controlling for temporal and intraindividual stability. Notably, such designs would likely have more data collection points and/or longer time lags with more measurement waves than employed in the current study, given habits for complex behaviors have been observed as taking longer to form than those for simple behaviors (Lally et al., 2010). Similarly, comparisons between complex and non‐complex behaviors should be interpreted with caution, given differences in complexity were assessed via differing behaviors in independent samples rather than direct manipulation within a homogenous population. Such a consideration may be important as while grouping complexity by researcher ratings is not uncommon (Greenwald et al., 2009; Hagger et al., 2023; Phipps et al., 2024), notable variability in complexity for any given behavior between individuals may exist (Dorina et al., 2024); and it is also plausible that differences in effects between samples could be due to differences in sample characteristics (e.g., pregnant women, undergraduate students, general population) and behaviors rather than behavioral complexity itself. Randomized controlled designs such as experiments and interventions may permit better causal inferences. In keeping our prior suggestions, such studies may manipulate self‐control, behavioral complexity, and habit formation and examine main an interactive effects on behavioral outcomes.

Second, all three samples employed self‐reported measures of behavior, habit, and self‐control. While there is evidence supporting the validity of self‐report behavioral measures (Godin, 2011; Hamilton et al., 2012), it would still be prudent for future research to replicate the current findings using non‐self‐report measures (e.g., using observation or devices). Similarly, habit was assessed using a meta‐cognitive measure of habit for each behavior. It is important to note that given individuals may not always be acutely aware of their automatic actions (Wood, 2016), such measures should be taken as inferences of habit rather than direct measures. Further, the habit measures employed in the current study referred to habit for each behavior in a general sense. It is also possible that results may differ when assessing habit as differing elements of the behavior, such as initiation or execution. Further, while we employed a commonly used trait self‐control measure and factor structure with previous validation (de Ridder et al., 2011), the inhibitory and initiatory components displayed sub‐optimal internal consistency in two of the three samples. In the current findings, the potential impacts of this poor internal consistency are partially mitigated by the use of PLS‐SEM, which accounts for measurement error within model estimates (Hair et al., 2017), reducing the effects of bias stemming from poor reliability (Hair & Sarstedt, 2019). However, even after attempting to correct for reliability issues using modeling, it is important to consider that these effects may still be at risk of attenuated or biased effects due to measurement or other forms of error introduced into the model by poor reliability (Nimon et al., 2012), and results, particularly where null effects have been observed, should be considered with potential attenuating effects in mind. Further, as the observed poor reliability finding is consistent with prior research on self‐control measurement, this may add to calls for modified measures with better psychometric integrity (Hagger et al., 2021; Maloney et al., 2012; Morean et al., 2014).

For the implications of the current study, it is also important to consider that we assessed the effects of trait self‐control, rather than state self‐control. While trait self‐control is generally a more researched construct in scientific literature, it is also difficult to target directly via intervention for lasting change (Inzlicht & Roberts, 2024). In terms of the potential implications of the current findings to intervention, it may also be important to assess how more directly malleable state self‐control and related self‐regulatory processes may influence the deployment of habits and automatic processes in pursuit of health behavior (e.g., Phipps et al., 2022). However, given potential temporal variability in the use of state self‐control‐related skillsets, this may be best assessed using more intensive N‐of‐1 or ecological momentary assessment strategies rather than the group‐level design employed in the current study.

Last, it is important to note that all three samples experienced higher‐than‐expected levels of attrition between measurement occasions, leading to smaller‐than‐desirable final sample sizes. While PLS‐based modeling has often shown accurate results even in more modest samples, it is nonetheless important to note that the replication of these findings in larger samples would yield more precise parameter estimates and associated confidence intervals. This is of particular importance given interaction effects, like the ones tested in the current research, are often theorized to be more sensitive to issues of statistical power than other forms of regression‐based tests (Cohen, 1988). Thus, despite the study having adequate power as per recommendations (Hair et al., 2017; Jhantasana, 2023), null effects should be treated with due caution. Further, it is also vital to note that significant attrition may lead to bias as individuals retained may represent those with greater motivation or control than those lost to attrition. Although our attrition analysis indicating little or no differences between the reported and baseline samples may somewhat allay these concerns. It should also be noted that we did not recruit our sample using randomized methods or stratify along key demographic characteristics, so findings should not be considered representative of the populations from which they were sampled. Future research may seek to adopt more pro‐active and intensive sampling strategies to minimize attrition and replicate these findings in samples with greater representativeness.

Taken together, the implications of this study paint a potentially interesting and largely novel picture of how self‐control may interact and influence habit and the enaction of behavior as habitual. Such a finding has implications for the understanding of habitual behavior and for psychological theories which attempt to explain the effects of habit and self‐regulation on behavior (e.g., temporal self‐regulation theory; Dorina et al., 2023; Hall & Fong, 2007), providing some preliminary evidence that the relationship may be more complex or context‐dependent than is often considered in prominent models. However, the limitations of the data mean that any interpretation of results is inherently speculative, and thus the current research should be viewed as exploratory and question generative, rather than as definitive assertions as to the interplay between habits and self‐control in complex as compared to simple behaviors.

## CONCLUSIONS

The current study sought to test some theory‐based relations between self‐control, habit, and health behaviors varying in complexity. Specifically, we investigated the effects of the initiatory and inhibitory components of self‐control on habit and of habit on health behavior, the role of habit as a mediator of self‐control component effects on health behavior, and the self‐control components moderating effect on the habit‐behavior relationship. We found direct effects of initiatory and inhibitory self‐control on habit and direct effects of habit on behavior in all three behaviors, reflected in non‐zero indirect effects of inhibitory self‐control on health behavior mediated by habit in two of the three behaviors. The inhibitory self‐control component moderated effects of habit on bootcamp attendance and physical activity behavior, but not on dental flossing, while the initiatory component exhibited no moderating effects. Findings provide preliminary evidence for the association of both inhibitory and initiatory self‐control with habit regardless of behavioral complexity, but the moderating effect of self‐control on the habit‐behavior relation is confined to behaviors classified as higher in complexity. This suggests that self‐regulatory capacities afforded to individuals with high trait‐self‐control, particularly inhibitory capacities, determine the extent to which more complex behaviors are controlled by habit. Findings warrant corroboration in more representative samples and using designs to better infer the direction and cause of the effects.

## CONFLICT OF INTEREST

The authors have no conflicts of interest to declare.

## SUPPLEMENTARY MATERIALS AND OPEN DATA

Data, outputs, and scripts are available at https://osf.io/v79bf/.

Supplementary Materials: https://osf.io/v79bf/?view_only=425f3bb7188c4b579dd355da99e51394.

## ETHICS STATEMENT

All procedures were approved the the Griffith University Human Research Ethics Committee.

## Supporting information


**Appendix A:** Self‐Reported Scales.
Appendix B: Profile Analysis.
Appendix C: A Comparison of parameter estimates between each sample.

## Data Availability

Data, outputs, and scripts are available at https://osf.io/v79bf/.

## ENDNOTE

1 Due to experimenter error one item of the SRBAI was not administered in sample 2 (“*Performing the recommended level of physical activity is something I do without thinking*”). Given the high correlations between items this is unlikely to have a notable effect on results but should be considered a limitation and data interpreted in light of this error accordingly.
