# Supplementary material for: Evidence inhibitory self‐control moderates effects of habit on complex but not simple health behaviors
Source: Appl Psychol Health Well Being. 2024 Dec 22;17(1):e12642. doi: 10.1111/aphw.12642 (PMC11664030; doi:10.1111/aphw.12642)
Supplement: Supplementary file 1 — Appendix A: Self‐Reported Scales. Appendix B: Profile Analysis. Appendix C: A Comparison of parameter estimates between each sample. [file APHW-17-0-s001.docx]

# Appendix A: Self-Reported Scales

|  |  |
| --- | --- |
| **All Samples** | |
| **Initiatory Self-Control** |  |
| I am lazy | 5 = Strongly disagree to 1 = Strongly agree |
| I have trouble concentrating | 5 = Strongly disagree to 1 = Strongly agree |
| I am able to work effectively toward long-term goals. | 1 = Strongly disagree to 5 = Strongly agree |
| I often act without thinking through all the alternatives | 5 = Strongly disagree to 1 = Strongly agree |
| **Inhibitory Self-Control** |  |
| I am good at resisting temptation. | 1 = Strongly disagree to 5 = Strongly agree |
| I have a hard time breaking bad habits | 5 = Strongly disagree to 1 = Strongly agree |
| I do certain things that are bad for me, if they are fun | 5 = Strongly disagree to 1 = Strongly agree |
| I refuse things that are bad for me. | 1 = Strongly disagree to 5 = Strongly agree |
| Sometimes I can’t stop myself from doing something, even if I know it is wrong | 5 = Strongly disagree to 1 = Strongly agree |
| Pleasure and fun sometimes keep me from getting work done. | 5 = Strongly disagree to 1 = Strongly agree |
| **Bootcamp Attendance Sample** | |
| **Habit** |  |
| Attending bootcamp is something I do automatically | 1 = Strongly disagree to 7 = Strongly agree |
| Attending bootcamp is something I do without having to consciously remember | 1 = Strongly disagree to 7 = Strongly agree |
| Attending bootcamp is something I do without thinking | 1 = Strongly disagree to 7 = Strongly agree |
| Attending bootcamp is something I start doing before I realize I am doing it. | 1 = Strongly disagree to 7 = Strongly agree |
| **Behavior** |  |
| Think about the past 4 weeks. In general, how often did you attend bootcamp? | 1 = Never, to 7 = Always |
| Think about the past 4 weeks. In general, to what extent did you attend bootcamp? | 1 = Never, to 7 = A large extent |
| **Physical Activity Sample** | |
| **Habit** |  |
| Performing the recommended level of physical activity is something I do automatically | 1 = Strongly disagree to 7 = Strongly agree |
| Performing the recommended level of physical activity is something I do without having to consciously remember | 1 = Strongly disagree to 7 = Strongly agree |
| Performing the recommended level of physical activity is something I start doing before I realize I am doing it. | 1 = Strongly disagree to 7 = Strongly agree |
| **Flossing Sample** | |
| **Habit** |  |
| Flossing is something I do automatically | 1 = Strongly disagree to 7 = Strongly agree |
| Flossing is something I do without having to consciously remember | 1 = Strongly disagree to 7 = Strongly agree |
| Flossing is something I do without thinking? | 1 = Strongly disagree to 7 = Strongly agree |
| Flossing is something I start doing before I realise I am doing it? | 1 = Strongly disagree to 7 = Strongly agree |
| **Behavior** |  |
| In the last week, how often did you floss? | 1 = Never, to 7 = Very Often |
| In the last week to what extent did you floss? | 1 = Never to 7 = A large extent |

# Appendix B: Profile Analysis

| Habit Split | Low Inhibitory Self Control | High Inhibitory Self-Control |
| --- | --- | --- |
| Strong Bootcamp Habits | 14 | 21 |
| Weak Bootcamp Habits | 20 | 14 |
|  |  |  |
| Strong Physical Activity Habits | 41 | 63 |
| Weak Physical Activity Habits | 62 | 41 |
|  |  |  |

# Appendix C: A Comparison of parameter estimates between each sample

|  | β1 | *SE1* | β2 | *SE2* | *t* | *df* | *p* | *d* |
| --- | --- | --- | --- | --- | --- | --- | --- | --- |
| **Bootcamp vs. Flossing** | Bootcamp | | Flossing | |  |  |  |  |
| Inhibitory Self Control → Behavioral Automaticity | 0.175 | 0.087 | 0.127 | 0.053 | 0.47 | 123.27 | .638 | 0.08 |
| Inhibitory Self Control → Behavior | 0.274 | 0.087 | 0.124 | 0.053 | 1.47 | 123.27 | .143 | 0.27 |
| Initiatory Self Control → Behavioral Automaticity | 0.274 | 0.087 | 0.167 | 0.053 | 1.05 | 123.27 | .296 | 0.19 |
| Initiatory Self Control → Behavior | -0.015 | 0.087 | 0.086 | 0.053 | -0.99 | 123.27 | .323 | -0.18 |
| Behavioral Automaticity → Behavior | 0.289 | 0.087 | 0.632 | 0.053 | -3.37 | 123.27 | .001 | -0.61 |
| Inhibitory Self Control by Behavioral Automaticity → Behavior | 0.303 | 0.087 | -0.006 | 0.053 | 3.03 | 123.27 | .003 | 0.55 |
| Initiatory Self Control by Behavioral Automaticity → Behavior | 0.076 | 0.087 | 0.077 | 0.072 | -0.01 | 171.43 | .993 | 0.00 |
| Inhibitory Self Control → Behavioral Automaticity → Behavior | 0.051 | 0.061 | 0.08 | 0.037 | -0.41 | 122.77 | .685 | -0.07 |
| Initiatory Self Control → Behavioral Automaticity → Behavior | 0.079 | 0.061 | 0.106 | 0.037 | -0.38 | 122.77 | .706 | -0.07 |
| Inhibitory Self Control → Behavior | 0.325 | 0.087 | 0.204 | 0.053 | 1.19 | 123.27 | .237 | 0.21 |
| Initiatory Self Control → Behavior | 0.064 | 0.087 | 0.192 | 0.053 | -1.26 | 123.27 | .211 | -0.23 |
| **Physical Activity vs. Flossing** | Physical Activity | | Flossing | |  |  |  |  |
| Inhibitory Self Control → Behavioral Automaticity | 0.315 | 0.072 | 0.127 | 0.053 | 2.10 | 239.35 | .037 | 0.27 |
| Inhibitory Self Control → Behavior | 0.088 | 0.072 | 0.124 | 0.053 | -0.40 | 239.35 | .688 | -0.05 |
| Initiatory Self Control → Behavioral Automaticity | 0.277 | 0.072 | 0.167 | 0.053 | 1.23 | 239.35 | .220 | 0.16 |
| Initiatory Self Control → Behavior | 0.081 | 0.072 | 0.086 | 0.053 | -0.06 | 239.35 | .955 | -0.01 |
| Behavioral Automaticity → Behavior | 0.298 | 0.072 | 0.632 | 0.053 | -3.74 | 239.35 | <.001 | -0.48 |
| Inhibitory Self Control by Behavioral Automaticity → Behavior | 0.223 | 0.072 | -0.006 | 0.053 | 2.56 | 239.35 | .011 | 0.33 |
| Initiatory Self Control by Behavioral Automaticity → Behavior | 0.053 | 0.072 | 0.077 | 0.072 | -0.24 | 314.35 | .814 | -0.03 |
| Inhibitory Self Control → Behavioral Automaticity → Behavior | 0.094 | 0.051 | 0.08 | 0.037 | 0.22 | 236.11 | .824 | 0.03 |
| Initiatory Self Control → Behavioral Automaticity → Behavior | 0.083 | 0.051 | 0.106 | 0.037 | -0.37 | 236.11 | .715 | -0.05 |
| Inhibitory Self Control → Behavior | 0.175 | 0.072 | 0.204 | 0.053 | -0.32 | 239.35 | .746 | -0.04 |
| Initiatory Self Control → Behavior | 0.171 | 0.072 | 0.192 | 0.053 | -0.23 | 239.35 | .814 | -0.03 |
| **Physical Activity vs. Bootcamp** | Bootcamp | | Physical Activity | |  |  |  |  |
| Inhibitory Self Control → Behavioral Automaticity | 0.175 | 0.087 | 0.315 | 0.072 | -1.24 | 150.84 | .217 | -0.20 |
| Inhibitory Self Control → Behavior | 0.274 | 0.087 | 0.088 | 0.072 | 1.65 | 150.84 | .102 | 0.27 |
| Initiatory Self Control → Behavioral Automaticity | 0.274 | 0.087 | 0.277 | 0.072 | -0.03 | 150.84 | .979 | 0.00 |
| Initiatory Self Control → Behavior | -0.015 | 0.087 | 0.081 | 0.072 | -0.85 | 150.84 | .397 | -0.14 |
| Behavioral Automaticity → Behavior | 0.289 | 0.087 | 0.298 | 0.072 | -0.08 | 150.84 | .937 | -0.01 |
| Inhibitory Self Control by Behavioral Automaticity → Behavior | 0.303 | 0.087 | 0.223 | 0.072 | 0.71 | 150.84 | .480 | 0.12 |
| Initiatory Self Control by Behavioral Automaticity → Behavior | 0.076 | 0.087 | 0.053 | 0.072 | 0.20 | 150.84 | .839 | 0.03 |
| Inhibitory Self Control → Behavioral Automaticity → Behavior | 0.051 | 0.061 | 0.094 | 0.051 | -0.54 | 151.99 | .589 | -0.09 |
| Initiatory Self Control → Behavioral Automaticity → Behavior | 0.079 | 0.061 | 0.083 | 0.051 | -0.05 | 151.99 | .960 | -0.01 |
| Inhibitory Self Control → Behavior | 0.325 | 0.087 | 0.175 | 0.072 | 1.33 | 150.84 | .186 | 0.22 |
| Initiatory Self Control → Behavior | 0.064 | 0.087 | 0.171 | 0.072 | -0.95 | 150.84 | .345 | -0.15 |
